# Developments in Neuroprotection for HIV-Associated Neurocognitive Disorders (HAND)

**DOI:** 10.1007/s11904-022-00612-2

**Published:** 2022-07-22

**Authors:** Dennis L. Kolson

**Affiliations:** Department of Neurology, University of Pennsylvania, Room 280C Clinical Research Building, 415 Curie Boulevard, Philadelphia, PA 19104 USA

**Keywords:** HIV-associated neurocognitive disorders, HAND, Neuroprotection, HIV, Treatment, Brain

## Abstract

**Purpose of Review:**

Reducing the risk of HIV-associated neurocognitive disorders (HAND) is an elusive treatment goal for people living with HIV. Combination antiretroviral therapy (cART) has reduced the prevalence of HIV-associated dementia, but milder, disabling HAND is an unmet challenge. As newer cART regimens that more consistently suppress central nervous system (CNS) HIV replication are developed, the testing of adjunctive neuroprotective therapies must accelerate.

**Recent Findings:**

Successes in modifying cART regimens for CNS efficacy (penetrance, chemokine receptor targeting) and delivery (nanoformulations) in pilot studies suggest that improving cART neuroprotection and reducing HAND risk is achievable. Additionally, drugs currently used in neuroinflammatory, neuropsychiatric, and metabolic disorders show promise as adjuncts to cART, likely by broadly targeting neuroinflammation, oxidative stress, aerobic metabolism, and/or neurotransmitter metabolism. Adjunctive cognitive brain therapy and aerobic exercise may provide additional efficacy.

**Summary:**

Adjunctive neuroprotective therapies, including available FDA-approved drugs, cognitive therapy, and aerobic exercise combined with improved cART offer plausible strategies for optimizing the prevention and treatment of HAND.

## Introduction

The persistence of neurocognitive impairment (NCI) associated with HIV infection, known by the Frascati criteria as HIV-associated cognitive disorders (HAND), or more generally as HIV-NCI, in the era of suppressive combination antiretroviral therapy (cART) is a challenge for people with HIV (PWH) [1, 2]. Although suppression of HIV replication reduces the risk for severe HIV-NCI, the effects of comorbid conditions, viral “blipping” or escape, chronic neuroinflammation and oxidative stress, and persistent effects of early injury in acute HIV infection (before cART achieves full suppression) probably contribute to long-term HIV-NCI risk [3, 4•]. Complete neuroprotection and reduction of HIV-NCI risk has not yet been achieved [5•]. Because HIV replication is the initiating factor for HIV-NCI, neuroprotection approaches must include complete suppression of HIV replication and HIV gene expression with modified cART regimens, and also modulation of cell/virus trafficking and spread, disordered metabolism, and inappropriate activation of oxidative stress and inflammation pathways that impact neuronal cell function.

Among comprehensive studies of the prevalence of HIV-NCI is the CNS HIV Antiretroviral Therapy Effects Research (CHARTER) cohort study [6]. Cognitive impairment (all causes) was found in 814 (52%) of 1555 (PWH) attending treatment centers in the USA [6]. Since the introduction of cART, the prevalence of severe HIV-NCI (HIV-associated dementia) has been reduced from approximately 20% in the pre-ART era to approximately 2–5% [6]. This reduction of HIV-NCI severity by cART represents the only consistent treatment success to date (reviewed in [7]). Unfortunately, less severe HIV-NCI is nonetheless associated with disabling functional impairment in activities of daily living in 10–12% of PWH [2, 6]. This morbidity burden will likely grow as the population of PWH ages, emphasizing a need for additional treatment strategies to improve brain protection in PWH.

**Fig. 1:**
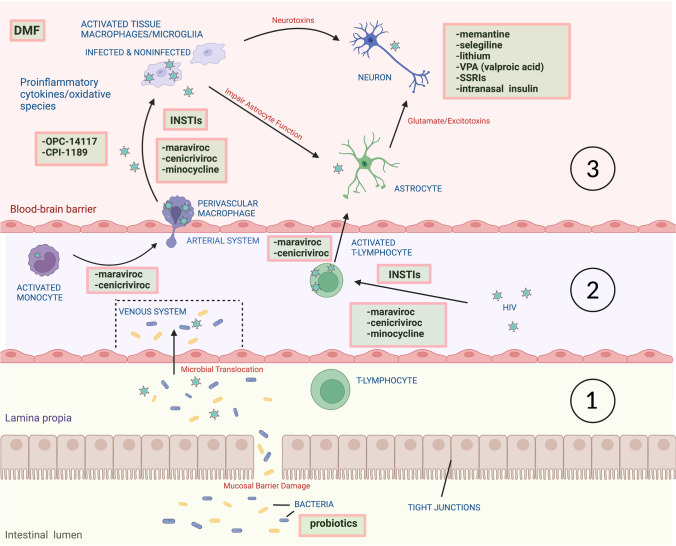
Proposed sites of neuroprotective drug effects in HIV neuropathogenesis. Multiple steps in HIV neuropathogenesis are potentially targetable for neuroprotection. Acute HIV infection results in (1) structural damage to the gut mucosal barrier and translocation of immune-activating microbial products into the systemic circulation, (2) transendothelial migration of HIV-infected immune cells into the CNS, and (3) HIV replication within perivascular/parenchymal macrophages and microglia and trafficking T lymphocytes. Amplification of pro-inflammatory and oxidative processes in infected and non-infected activated glial cells is associated with release of toxic metabolic products, including reactive oxygen species, pro-inflammatory cytokines, HIV proteins, and excitotoxic neurotransmitters. *Abbreviations:* INSTIs—integrase strand transfer inhibitors; DMF—dimethyl fumarate; SSRIs—selective serotonin re-uptake inhibitors

This review discusses neuroprotective strategies to improve neurocognitive outcomes in PWH, for which the foundation is suppressive cART (Table 1, Fig. 1). More effective neuroprotection strategies will require the continued evolution of cART regimens and the development of adjunctive therapies. Many preliminary successes in reducing HIV-NCI have been seen in small pilot clinical trials, which have often later been recognized as treatment failures when examined in larger well-controlled validation studies. Nonetheless, as knowledge of the functional, biochemical, and structural integrity of the CNS in PWH continues to grow, and as new neuroprotective/neuromodulating medications and drug delivery systems are developed for other CNS disorders, future success seems likely. This review will target selected areas of cART strategies, therapies for neuroinflammation and oxidative stress, neurodegenerative disease therapies, neuropsychiatric medications, trophic factors, and exercise therapy, with the recognition that some areas of potential interest are outside of the selected scope of this review.

**Table 1:** Clinical studies of adjunctive therapies for HIV-associated neurocognitive disorders

Treatment	Molecular target/action	Proposed effects	Clinical data	References
Modified cART regimens				
Maraviroc	CCR5 receptor blocker	Block HIV entry; block CCR5 receptor signaling	Open-label 15-patient maraviroc intensification of cART showed neurocognitive improvement over 24 weeks; Open-label 14-patient maraviroc intensification study showed neurocognitive improvement at 6 and 12 months	[24] [25]
Cenicriviroc	CCR5 and CCR2 receptor blocker	Block HIV entry and CCL2-mediated monocyte chemotaxis	Open-label 17-patient study of cenicriviroc intensification of cART showed modest neurocognitive improvement over 24 weeks	[29•]
Dolutegravir (DTG)	Integrase strand transfer inhibitor (INSTI)	Block final ligation (integration) of HIV provirus into cellular genome	Cross-sectional 202-patient study of DTG inclusion in cART associated with worse neurocognitive improvement and lower brain volume; Longitudinal 254 patient study up to 37 weeks showed switch to DTG associated with worsened depression; practice effects obscured NP test results; 30 patient study initiating INSTI < 3 months vs > 6 months after HIV acquisition showed no difference in neurocognitive performance after 48 weeks RCT of 191 patients with ART intensification with DTG alone or with maraviroc showed no significant effect on neurocognitive improvement over 96 weeks. DTG treatment had no adverse neuropsychiatric effects	[32•] [34••] [33•] https://www.natap.org/2022/CROI/croi_187.htm)
Anti-inflammatory/antioxidant				
OPC-14117	Free radical scavenger	Decrease oxidative injury by free radicals	Phase I/II study in 30 patients with mild HIV-NCI for 12 weeks, neurocognitive improvement did not reach significance	[5•]
CPI-1189	TNFa	Inhibit p38 MAPK and inhibit TNFa pro-inflammatory signaling; antioxidant properties	RCT of 64 patients with mild-moderate HIV-NCI treated for 10 weeks; no neurocognitive improvement	[5•]
Memantine	NMDA receptor antagonist	Block excessive calcium influx through NMDA receptor channel; increase BDNF levels	RCT of 98 patients with mild HIV-CI treated for 20 weeks, with open-label extension up to 60 weeks; Modest preservation of NAA levels (neuronal integrity) by MR spectroscopy; no neurocognitive improvement	[93, 94]
Selegiline	MAO-B Inhibitor	May reduce antioxidant burden of cell	RCT of 124 patients with mild – moderate HIV-CI treated for 24 weeks, with open-label extension up to 60 weeks; No neurocognitive improvement overall; some improvement in NP subtests	[97] [96]
Minocycline	5-Lipo-oxygenase and others	Decrease CCL2 levels in CSF, improve SIV encephalitis, suppress HIV replication and inhibit secretion of TNF-α, IFN-γ and IL-2 by lymphocytes	RCT of 107 patients with progressive mild – moderate HIV-NCI treated for 24 weeks; no neurocognitive improvement	[5•]
Statins	HMG-CoA reductase inhibitors	Weak anti-inflammatory, antioxidant effects	ACTG Longitudinal Linked Randomized Trials (ALLRT) cohort (3949 participants) showed no association between statin use and neurocognitive performance	[125]
Neuropsychiatric drugs				
Lithium	Inhibit dopamine, glutamate neurotransmission (NMDA receptor), promotes GABA-ergic transmission, induce BDNF, Bcl2 PKC, GSK-3β inhibitor	Reduce excitotoxicity, trophic effects	Open-label 21-patient study for 12 weeks showed improved neurocognitive performance; Open-label 15-patient study for 10 weeks showed no neurocognitive improvement; RCT of 61 patients with moderate – severe HIV-NCI treated for 24 weeks; no neurocognitive performance difference compared to placebo	[101] [102] [103]
Sodium valproate (VPA)			40-patient trial (8 VPA, 32 no VPA in advanced HIV) showed worse neurocognitive performance with VPA Placebo-controlled pilot study of 22 PWH patients (16 with HIV-NCI; 6 without HIV-NCI) showed ‘trend’ towards neurocognitive improvement	[104] [105]
Selective serotonin re-uptake inhibitors (SSRIs): citalopram sertraline trazadone paroxetine	Selective serotonin re-uptake inhibitors	Some (citalopram, sertraline, trazadone) associate with lower HIV viral levels in CSF	RCT of 45 patients subdivided to paroxetine and/or fluconazole for 24 weeks; in those receiving paroxetine, neurocognitive improvement in 4/6 tests, worsening in 2/6; citalopram, sertraline, trazadone use linked historically to better neurocognitive performance in PLWH	[111] [110] [106••] [107] [108•]
Trophic agents				
Intranasal insulin	Neuronal insulin receptor activation	Possible trophic effects in the olfactory bulb, hypothalamus, and cerebellum and areas involved in memory, including the hippocampus and limbic system	Placebo-controlled trial of 21 patients with mild-moderate HIV-NCI showed improvement in memory and attention at 12 and 24 weeks	https://www.natap.org/2021/CROI/croi_91.htm
Non-pharmacological alternative therapies				
Aerobic exercise	Metabolism, inflammation, angiogenesis, synaptogenesis	Reduce oxidative stress, inflammation, promote neurogenesis, angiogenesis, synaptogenesis	Longitudinal study of 291 PLWH with consistent physical activity (≥ 50% study visits) maintain better neurocognitive performance (4 domains) over 35 months vs. sedentary patients	[122•]
Cognitive training	Neuronal metabolism	Promotion of neuroplasticity to increase cognitive reserve	A review of past and current studies-in-progress suggesting improvement in neurocognitive domains targeted by specific training strategies in PLWH	[123•]

### Improving cART Regimens

Improving ART effectiveness in suppressing HIV replication in the CNS by either increasing penetration into the CNS or intensifying ART through addition of other classes of drugs is a strategy for reducing mild-moderate HIV-NCI risk. Improved penetration or retention of bioavailable ART drugs in the CNS might decrease CNS HIV replication and reduce concomitant release of neurotoxins from infected and activated macrophages/microglia and immune-activated astrocytes [8••, 9]. Furthermore, choosing ART regimens based upon their efficacy in reducing HIV replication in monocyte and macrophage lineages may be more efficacious in targeting HIV-NCI pathogenesis [10].

### Maintaining Viral Control: the Problem of Cerebrospinal Fluid Viral “blipping”

Despite the general effectiveness of cART in suppressing HIV replication, a surprisingly high rate (10–20%) of HIV “blipping” (defined as spontaneous expression of HIV RNA copies in CSF in the setting of undetectable viral RNA in plasma) is observed [11]. This suggests that cART does not maintain consistent long-term viral suppression within the CNS reservoir, and that “blipping” might be a risk factor for persistent HIV-NCI risk in virally suppressed PWH [12]; thus, development of new, more consistently suppressive cART regimens may be required for more effective neuroprotection [13]. The suppression of HIV blipping should be considered as a goal for improving cART efficacy in reducing risk for HIV-NCI, through enhancing CNS ART delivery.

### Enhancing ART CNS Penetrance

Classification of ART drugs by the CNS penetrance efficacy (CPE) ranking has been developed to rank individual drugs according to their ability to penetrate into the CNS [14, 15]. Although application of favorable CPE-ranked ART regimens has shown promise in reducing CSF expression of markers of neuroinflammation, CSF HIV load, and incidence or progression of HIV-NCI in patients in some studies, enhanced efficacy of cART regimens based upon the CPE has not been consistently observed [16, 17, 18••]. However, cumulative evidence does associate some high-CPE regimens with higher CNS HIV suppression and a possible benefit for HIV-NCI, thus supporting additional, larger-scale investigations [18••].

The inception of nanoparticulate-ART (nanoART), ART nanosuspensions, and associated monocyte/macrophage targeted ART strategies has also produced promising results, at least in humanized HIV-infected mice, which suggests that enhancing CNS ART delivery can have neuroprotective effects [19–21]. These and other re-formulated ART preparations that produce longer-lasting effective drug release may soon be ready for new human clinical trials for HIV-NCI neuroprotection [22, 23].

### ART Intensification with Chemokine Receptor Blockade

The chemokine receptor CCR5, which is expressed at high levels on activated T lymphocytes and macrophages, serves as an HIV-1 co-receptor and regulator of chemotaxis. Maraviroc, a CCR5 chemokine receptor antagonist and entry inhibitor, has been investigated as an ART intensification drug for reduction of HIV-NCI risk, with promising early results [24, 25]. Although a large-scale maraviroc intensification study for HIV-NCI risk reduction has not been developed, there is enthusiasm for targeting CCR5 for treatment in neuroinflammatory disorders in general, and further investigations of maraviroc as a neuroprotectant in HIV-NCI could be justified [26••].

In contrast, cenicriviroc is a blocker of CCR5 and CCR2 chemokine receptors, thereby not only blocking HIV entry into cells, but also inflammation and monocyte migration [27]. CCL2 (the cognate ligand for CCR2) is a chemotactic chemokine that recruits CD14 + CD16 + monocytes across the blood–brain barrier. CCL2 is elevated in the CNS of HIV-infected individuals with HIV-NCI, even those treated with ART (reviewed in [28]). A pilot 24-week trial of cenicriviroc in 17 cART-suppressed PWH with mild to moderate HIV-NCI showed some modest beneficial effect [29•]. Thus, the potential neuroprotective effects of combination blockade of chemokine receptors (CCR5, CCR2) involved in HIV entry and those involved with chemotaxis should be further considered in larger prospective studies.

### Integrase Strand Transfer Inhibitors (INSTI)

Integrase strand transfer inhibitors (INSTI) can effectively block the final ligation (integration) of the HIV provirus into cellular DNA, thereby limiting productive HIV replication. INSTI are widely used, effective, and generally well-tolerated, although weight gain may be associated with their use [30••, 31•]. In recent studies, inclusion of INSTI, including dolutegravir (DTG), in cART regimens in early HIV treatment did not result in improved neurocognitive performance or neuroimaging assessments of brain integrity [32•, 33•, 34••]. Additionally, a recent 96-week, randomized, placebo-controlled intensification study (ACTG 5234; 25 national and international sites; ClinicalTrials.gov Identifier: NCT02519777) examined the addition of DTG with or without maraviroc to suppressive ART. Unlike previous studies, this study included a DTG arm alone, and it was randomized, double-blinded, and placebo-controlled. The study showed no significant difference from placebo effects on neurocognitive performance in 191 PWH (https://www.natap.org/2022/CROI/croi_187.htm). Notably, there was no worsening of symptoms of depression on those individuals receiving DTG alone, which is of considerable interest in view of reports of adverse neuropsychiatric effects of DTG [34••, 35]. Thus, although no new ART-improvement INSTI strategies that consistently increase ART efficacy in reducing HIV-NCI risk have been developed, there is need for further study of INSTI. Overall, the strategy of increasing cART CNS effectiveness and reducing CNS ART toxicity risks are worthy goals to develop as newer ART drugs of all classes are brought forth.

### ART Toxicity

The possibility of long-term ART neurotoxicity is inescapable, and reducing such risk without sacrificing ART suppressive efficacy is challenging [35]. Long-term treatment with ART is associated with a range of systemic toxicities including hepatic steatosis, peripheral neuropathy, cardiomyopathy, pancreatitis, ototoxicity, retinopathy, and lipodystrophy. Substantial toxicity of various nucleoside reverse transcriptase inhibitors (NRTIs) in different tissue types has been observed, and ART toxicity within the CNS compartment is a major concern. In vitro studies demonstrate that numerous ART drugs are directly neurotoxic at concentrations detected in the CSF of PLWH on cART, and certain cART regimens, including those with INSTI, have been associated with poorer neurocognitive outcomes [35–37]. Concerns over the potential risk for dolutegravir (DTG) use during pregnancy as an inducer of neural tube defects have proved to be unfounded, however [38••, 39••]. Continual surveillance of cognitive functioning in PLWH as cART regimens evolve is essential for defining and ultimately mitigating toxicity risks, which are clearly superseded by the neuroprotective effects of cART.

## Inflammation and Oxidative Stress in HIV-NCI

### Neuroinflammation

In ART-naïve subjects, HIV infection of the brain produces robust expression of pro-inflammatory cytokines and chemokines, reactive oxygen species, glutamate and other non-proteinaceous neurotoxins, and perhaps some HIV proteins; each of these has been linked to disrupted neuronal function and architecture [40, 41]. Inflammatory mediators modulate the permissibility of the blood–brain barrier and the entry of infected lymphocytes and monocytes into the CNS. Additionally, inflammatory processes in the periphery, such as gut permeability and bacterial translocation, have been linked to HIV-NCI [42, 43]. Therefore, reducing inflammation in the periphery as well as within the CNS could be expected to reduce HIV-NCI risk in PLWH. However, consistency of expression of these inflammation factors and their associated linkage to neuronal injury and dysfunction in individuals on suppressive cART is not as clear, and this thesis has been challenged [44]. Regardless of whether persistent neuroinflammation and/or intermittent neuroinflammation associated with HIV CSF blipping in cART-suppressed patients drive HIV-NCI pathogenesis, each should be further investigated both in PWH and in non-human primate models of suppressed SIV infection to meet this challenge [45].

### Oxidative Stress

Oxidative stress is considered to be an imbalance between oxidants and antioxidant defenses, and it manifests as oxidative damage to proteins, nucleic acids, and other biomolecules, with resulting cellular injury. Oxidative stress and inflammation are closely related processes that often co-exist in chronic diseases, and neuroprotection strategies should target both processes concurrently [46]. HIV infection induces pathological oxidative stress, as evidenced by diminished levels of reduced glutathione in plasma, lymphocytes, and PBMC and elevated levels of biomarkers of lipid peroxidation products such as malondialdehyde and hydroperoxide; several of these and other markers of oxidative stress correlate with disease progression and mortality [47–51]. Oxidative stress can in turn drive inflammation through enhancing NF-κB-driven HIV replication and release of proinflammatory cytokines [52–55].

Among the damaging molecules involved in oxidative injury are free radicals (superoxide anion, nitric oxide, hydroxyl radical), reactive oxygen species (including free radicals and non-radicals such as H_2_O_2_ and peroxynitrite), and reactive nitrogen species [46, 56]. Suppression of HIV replication with cART significantly reduces immune activation and oxidative stress; however,this suppression is incomplete [57–59]. This suggests that the pathways driving production of neurotoxins remain active and that neuronal damage can accumulate even during virologic suppression. Despite some previous therapeutic failures in treating HIV-NCI with free radical scavenging drugs, newer drugs with broader targeting of inflammation and oxidative stress pathways may offer new options worth pursuing [8••].

### Contribution of Systemic Inflammation: the Gut

HIV infection is associated with translocation of microbial products across a damaged gastrointestinal tract and dysbiosis of bacterial populations, although a recent study has questioned the association between HIV infection and altered gut mucosal integrity in PLWH [60–63]. Decreased microbial diversity and alterations of abundance of different genera, including increased abundance of the genus *Prevotella* and decreased prevalence of the genus *Bacteroides*, are characteristic of HIV infection [62]. Generally, changes towards decreased abundance of commensal, protective bacteria and increased abundance of pro-inflammatory bacteria are observed, with many variations in specific genera. Increased systemic inflammation has been linked to the translocated microbial product LPS, which is elevated in the plasma, but not the CSF, of PWH [64•]. Plasma LPS associates with monocyte activation, indicated by elevated blood levels of soluble CD14 (sCD14) and sCD163, and elevated expression of monocyte-associated CD14 and CD16 [65]. Elevated blood levels of CD16 + monocytes, sCD14, sCD163 and total CD14 + monocyte HIV DNA content associated with microbial translocation have been correlated with an increased risk for HIV-NCI. Therefore, the gut microbiome and LPS are considered targets for reducing pathogenic effects of microbial translocation [66].

Although targeting of LPS (e.g., through sequestration with the drug sevelamer) has not demonstrated benefits for cardiovascular dysfunction associated with inflammation in PWH, targeting LPS has not yet been explored in HIV-NCI risk reduction. Nonetheless, it should be considered [62]. Also, therapies targeted towards directly reducing microbial translocation (e.g., preserving gut mucosal integrity) in PLWH may have a role in neuroprotection, although no such specific therapies currently exist [67]. Focusing on correcting the gut microbiome dysbiosis as a means of promoting gut integrity and additionally reducing systemic inflammation is a reasonable strategy for attempting to reduce HIV-NCI risk; indeed, preliminary evidence suggests that probiotic dietary supplementation may reduce neuroinflammation and promote neurocognitive recovery in PLWH [67–69].

### Targeting Oxidative Stress and Associated Inflammation

To date, results of clinical trials with agents that, to varying degrees, target inflammation with or without oxidative stress [OPC-14117 (free radical scavenger), CPI-1189 (TNFα blocker), selegiline (MAO-B inhibitor), minocycline (antibiotic, antioxidant), paroxetine (SSRI), lexipafant (platelet activating factor receptor blocker), maraviroc (CCR5 blocker)] as a strategy for reducing HIV-NCI risk have failed to clearly confirm benefits [5•, 8••, 70••]. Nonetheless, consistent evidence for chronic inflammation, which is linked to oxidative stress in multiple body compartments in PLWH, supports a rationale for continuing efforts to test more robust therapeutics for reducing chronic inflammation (in combination with oxidative stress) to lower the risk for HIV-NCI [71]. Previous treatment failures with agents that are either relatively highly selective in their targets, or relatively weakly effective, suggests that broader targeting of inflammatory and oxidative pathways with broader-spectrum agents should be considered, particularly those already effectively used to treat other neuroinflammatory diseases.

### Broadening the Target for Better Efficacy: the Host Antioxidant (ARE-driven) Response

The host protective antioxidant response is mediated in part through activation of the transcription factor NF-E2-related factor 2 (Nrf2), which drives the antioxidant response element (ARE), a cis-acting regulatory sequence in the promoter region of numerous antioxidant and anti-inflammatory genes [72]. Among these Nrf2 targets are several antioxidant enzymes, including heme oxygenase-1 (HO-1), an inducible, detoxifying enzyme that is critical for limiting oxidative stress, inflammation, and cellular injury within the CNS and other tissues. Transcriptional regulation of HO-1 depends partly upon *HMOX1* promoter region genetic variations, including a (GT)_*n*_ dinucleotide repeat, which associate higher basal *HMOX1* transcriptional activity and inducibility, and better outcomes in inflammatory and oxidative stress–associated diseases [72–74]. We identified HO-1 as a key marker and potential regulator of HIV-NCI, and further showed that the presence of one or more short HO-1(GT)_*n*_ repeat alleles, which are known to have higher HO-1 transcriptional activity, associate with a decreased risk for HIV neuroinflammation and HIV NCI [75–78, 79•]. Furthermore, our studies suggest that African-Americans may be more vulnerable to HIV NCI than others because of differences in their HO-1 (GT)_*n*_ allele genotype prevalence [79•]. Reduced risk for HIV-NCI and its associated neuroinflammation in PWH with common *HMOX1* genetic variations argues strongly for a role for HO-1, among other ARE-driven antioxidant enzymes, as appealing therapeutic targets for HIV-NCI risk reduction.

### Nrf2 Activators: Targeting the ARE Antioxidant Response

Therapeutic targeting of ARE-driven gene expression to enhance antioxidant responses and associated inflammation cascades has proved successful in the treatment of multiple sclerosis, and it is proposed as a strategy for prevention of HIV-NCI [71, 75, 80]. The Nrf2 transcriptional activator, dimethyl fumarate (DMF), is an FDA-approved treatment for multiple sclerosis, with excellent CNS penetration and efficacy in reducing neuroinflammation in demyelinating plaques, clinical relapse rates, and accumulation of new demyelinating lesions [81]. DMF, and its primary in vivo metabolite monomethyl fumarate (MMF), effectively induce HO-1 expression, inhibit NFκB nuclear translocation, and attenuate macrophage-mediated neurotoxicity in in vitro models of HIV neurotoxicity [82, 83]. Furthermore, DMF suppresses the Warburg effect (proinflammatory induction of aerobic glycolysis) in the mammalian brain, which may underlie its neuroprotective effect in multiple sclerosis patients [84••]. Daily oral delivery (> 100 days) of DMF to SIV-infected rhesus macaques resulted in concordantly increased expression of ARE-driven antioxidant enzymes (including HO-1) and reduced oxidative DNA and protein damage in multiple brain regions, compared with untreated, non-infected macaques [85•]. The efficacy of DMF in multiple sclerosis patients, in combination with in vitro and in vivo studies of HIV/SIV neuroprotection makes it an attractive candidate for HIV-NCI neuroprotection studies. Additionally, several other Nrf2-activating drugs (bardoxolone, resveratrol, others) are also being examined in multiple clinical trials for various inflammatory/oxidative disease states, further supporting Nrf2-directed therapeutics as a promising target for HIV-NCI neuroprotection trials [86•].

### Statins

Statins are routinely used as lipid-lowering agents that act via blocking hydroxymethlyglutaryl-CoA (HMG-CoA) enzymatic activity. They also express other pleiotropic anti-inflammatory, antioxidant, and immunomodulatory effects, which make them attractive for tissue protection in a variety of disease states [87•]. High-dose (80 mg daily) atorvastatin has been shown to reduce monocyte activation in cART-treated PLWH, but beneficial effects on HIV-NCI risk have not been observed [88, 89]. A review of randomized controlled trials and observational studies of various study designs not involving PLWH indicates that initiation of statin therapy late in life does not prevent cognitive decline or dementia over 3–5 ensuing years [90]. Although some studies have indicated a protective effect against acute brain injury, studies in multiple sclerosis patients (in which neuroinflammation and oxidative stress are major pathogenic mechanisms) have also failed to demonstrate protective effects of statins [91, 92•]. This stands in contrast to the protective effects of DMF in multiple sclerosis patients, which suggests that more robust induction of Nrf2-driven anti-inflammatory and oxidative stress pathways is necessary for neuroprotection. Thus, lacking any additional evidence for statin neuroprotective effects in humans, the likelihood of beneficial effects of statins on HIV-NCI risk appears low.

## Other Therapies Applied to Neurodegenerative Diseases and Neuropsychiatric Disorders

### Neurodegenerative Disease Therapies

Several aforementioned therapies used in neurodegenerative diseases (Alzheimer’s disease, Parkinson’s disease) are directed towards neuroprotection while others are directed towards treating symptoms. Memantine is approved in the treatment for Alzheimer’s disease and acts as a non-competitive NMDA glutamate receptor antagonist. A short-term clinical memantine trial in HIV-NCI patients provided neuroimaging evidence (magnetic resonance spectroscopy) of some preservation of neuronal integrity, but without significant neurocognitive improvement [93]. A longer-term follow-up failed to reveal a clinically demonstrable neurocognitive benefit, and so additional studies of memantine for HIV-NCI are probably not warranted [94].

Selegiline, a monoamine oxidase B (MAO-B) inhibitor used in the treatment of early-stage Parkinson’s disease, is proposed to act as a neuroprotectant by reducing the antioxidant burden of the cell [95]. However, clinical studies have shown neither a reduction in markers of oxidative stress nor improvement in cognitive performance with short-term (24 weeks) transdermal selegiline, thus arguing against additional selegiline efficacy trials for HIV-NCI [96, 97].

### Neuropsychiatric Drug Therapies

Among drugs used in the management of neuropsychiatric disorders, sodium valproate (VPA) and lithium are approved for treatment of bipolar disorder and related mood disorders; each inhibits glycogen synthase kinase-3β and provides neuroprotection against HIV-induced toxicity in vitro and in mouse models [98–100]. Although several open-label pilot studies of lithium therapy demonstrated improved neurocognitive performance in PLWH with HIV-NCI, a more recent randomized, placebo-controlled study of lithium showed no beneficial effect on HIV-NCI [101–103]. In an observational study of VPA use in PLWH over an average of 18 months, VPA users vs. non-users demonstrated poorer neurocognitive performance [104]. Subsequently, a placebo-controlled pilot study of VPA also failed to demonstrate cognitive improvement in PLWH and with HIV-NCI[105]. It is important to note that VPA is also a histone deacetylase inhibitor (HDAC), and thus there is at least a theoretical risk of reactivating HIV expression from a state of latency, which could be a potential confounder in identifying possible neuroprotective effects in vivo. Nonetheless, available clinical trial data to date do not convincingly support further trials for either lithium or VPA for HIV-NCI reduction risk.

Selective serotonin reuptake inhibitors (SSRIs) have gained increasing attention for their beneficial effects on depression and their relatively limited side effects in PLWH [106••, 107]. Additionally, some benefit of SSRIs in reducing immune activation may be seen. Within the Veterans Aging Cohort Study, investigators showed significant, albeit modest, reduction in plasma CD14 and IL-6 in SSRI users, among 1546 HIV-positive veterans [108•]. However, very limited research has been conducted to prospectively evaluate SSRIs as adjunctive therapies for HIV-NCI [109–111]. A recent double-blind, placebo-controlled study of 45 PWH sub-divided into patient groups receiving paroxetine and/or fluconazole over 24 weeks showed significant improvement in four neuropsychological tests (including the NPZ8 summary measure), with worse performance in two neuropsychological tests, in those receiving paroxetine [111]. Considering the results of this first-of-its kind, controlled study of SSRI and HIV-NCI outcomes in PLWH, additional SSRI studies should be considered. At least one such study has been proposed [112].

## Other Therapies Targeting Cell Trafficking and Trophic Factors

### Cell Trafficking

Limiting trafficking of HIV-infected cells into various tissue compartments, including the CNS, may be considered as a possible neuroprotective strategy, although this line of research is primarily directed towards limiting the size of the HIV reservoir in lymphoid tissues. Fingolimod (FTY720), an FDA-approved treatment for multiple sclerosis, is a lysophospholipid sphingosine-1 phosphate receptor modulator that limits egress of CD4 + and CD8 + T lymphocytes from lymph nodes [113•]. A pilot study in SIV-infected rhesus macaques showed reduction in SIV DNA-containing lymphocytes in lymph nodes in fingolimod-treated animals, presumably reflecting the action of retained nodal cytotoxic T lymphocytes [114•]. No studies of CNS effects of fingolimod in SIV-infected macaques have been published. Although fingolimod is currently used for treating multiple sclerosis, recent concerns about the possibility of reduced response to SARS CoV-2 vaccination may temper enthusiasm for expanding neuroprotection studies to HIV-NCI [115].

Natalizumab, an FDA-approved treatment for multiple sclerosis, is an anti-α4 integrin-targeting monoclonal antibody that inhibits monocyte and T-lymphocyte trafficking to the brain and other tissues [116, 117]. In the rhesus macaque model of multiple sclerosis (experimental autoimmune encephalomyelitis), natalizumab blocked inflammation, demyelination, and ingress of monocytes and T lymphocytes into the CNS [116]. In a pilot study of SIV infection of rhesus macaques, natalizumab treatment of prior to SIV inoculation prevented CNS SIV infection, while treatment post-SIV infection significantly reduced progressive SIV-associated neuronal injury [118]. These data suggest that natalizumab could have some neuroprotective effect against ongoing neuronal injury secondary to continuing immune cell trafficking into the CNS in PLWH. However, the relatively high-risk of reactivation of JC virus and associated genesis of progressive multifocal leukoencephalopathy with prolonged treatment with natalizumab, compared to other treatments in multiple sclerosis patients, will also likely reduce enthusiasm for neuroprotection studies in HIV-NCI.

### Trophic Agents Targeting Metabolism: Intranasal Insulin

To date, published studies of clinical investigations of trophic factors for the treatment of HIV-NCI are limited to the use of intranasal insulin. In patients without HIV, administration of insulin intranasally has been associated with improved cognitive performance in patients with diabetes mellitus, while a randomized clinical trial in patients with Alzheimer’s disease showed no benefit [119•, 120•]. A preliminary report of a pilot study (ClinicalTrials.gov Identifier: NCT03081117) of intranasal insulin administration for the treatment of HIV-NCI in 21 PWH indicated improvement in neuropsychological tests of memory and attention (https://www.natap.org/2021/CROI/croi_91.htm). A full report of clinical and biomarker outcomes in this pilot study is anticipated (Norman Haughey, personal communication).

## Alternative Adjunctive Therapies: Cognitive Therapy and Exercise

Non-pharmacologic interventions such as cognitive therapy and exercise may have a role in improving HIV-NCI through direct or indirect effects [121, 122•, 123•]. Cognitive training in PWH has demonstrated beneficial effects in specific cognitive domains that are targeted by the training, and an interventional clinical trial with individualized targeting of specific domains of deficit in 109 PWH has been completed [123•, 124] (ClinicalTrials.gov Identifier: NCT03122288). In general, the neuroprotective effects of aerobic exercise on structural brain integrity have been well-documented, but whether cognitive therapy also has a protective effect on brain structural integrity is not known [125, 126]. In PWH, physical exercise indeed associates with lower risk for HIV-NCI [122•, 127]. A longitudinal study of 291 PWH demonstrated that individuals with consistent physical activity (physical activity and ≥ 50% of study visits) maintained better neurocognitive functioning in domains of verbal fluency, working memory, speed of information processing executive function, and motor function over a mean period of 35 months, even when adjusting for confounding factors [122•]. Initiating cognitive training with regular aerobic exercise is a low-cost, low risk strategy that should be combined with adjunctive pharmacologic neuroprotection strategies in PWH for reducing risk for HIV-NCI.

## Conclusions

The persistent risk of HIV-NCI in PLWH despite suppressive cART requires improvement of cART CNS effectiveness and the implementation of adjunctive therapies for risk reduction based upon preservation of brain integrity. Lessons from past clinical trials suggest that early intervention with agents that more broadly target relevant pathogenic pathways effects should be prioritized, and among attractive targets are pathways of inflammation, oxidative stress, neurotransmitter metabolism, and metabolism-modulating trophic factors. Minimizing effects of impaired gut mucosal integrity and altered gut microflora with microbiome modifications also holds future promise. For drug therapies, it is likely that concurrent initiation of an adjunctive therapeutic drug in combination with cART in acute HIV infection may have the most profound effect, and even short-duration treatment may have long-term benefits. Agents that robustly activate endogenous antioxidant response genes while suppressing associated inflammatory responses should be pursued, and one example is dimethyl fumarate, widely used in the treatment of multiple sclerosis. Other currently approved neuromodulating drugs that limit immune cell trafficking into the CNS in multiple sclerosis patients are probably too risky for use in PWH. Re-purposing of other available drugs, including SSRIs such as paroxetine, which also directly or indirectly suppress inflammatory and oxidative stress processes, deserves immediate attention. Development of new pharmacological agents targeting these and other newly identified pathological pathways should also be pursued as a longer-term strategy. Each approach should be combined with aerobic exercising and cognitive therapy, which can be promoted by individualized lifestyle training. In total, these approaches will likely also mitigate effects of comorbidities that contribute to HIV-NCI and enhance the quality of life for PWH.
